# Meta-Optical Chirality and Emergent Eigen-polarization Modes via Plasmon Interactions

**DOI:** 10.1038/srep40718

**Published:** 2017-02-08

**Authors:** Matthew Moocarme, Nicholas V. Proscia, Luat T. Vuong

**Affiliations:** 1 The Graduate Center of CUNY, 365 5th Ave, New York, NY, 10016, USA; 2 Queens College of CUNY, 65-30 Kissena Blvd, Flushing Queens, NY, 11367, USA.

## Abstract

The response of an individual meta-atom is often generalized to explain the collective response of a metasurface in a manner that neglects the interactions between meta-atoms. Here, we study a metasurface composed of tilted achiral meta-atoms with no spatial variation of the unit cell that derives appreciable optical chirality solely from the asymmetric interactions between meta-atoms. The interactions between meta-atoms are considered to stem from the Lorentz force arising from the Larmor radiation of adjacent plasmonic resonators because their inclusion in a simple model accurately predicts the bonding/anti- bonding modes that are measured experimentally. We also experimentally observe the emergence of multiple polarization eigenmodes, among other polarization-dependent responses, which cannot be modeled with the conventional formalism of transmission matrices. Our results are vital to the precise characterization and design of metasurfaces.

Metamaterials are composed of sub-wavelength components, or meta-atoms that individually alter the intensity and phase of light. When placed on a 2D surface, metamaterials are known as metasurfaces^1^ and may contribute to the design of future quarter^2^, half^3^-wave plates, cross-polarizers^4^, and even ultra-thin lenses^5^,^6^. Chiral metasurfaces can ultimately be defined as those that exhibit a response dependent on the incident circular-polarization handedness, though the specific mechanism behind the phenomena has been debated; it has been argued that planar structures such as metasurfaces cannot exhibit true optical activity (OA) or circular dichroism (CD) without the excitation of magnetic resonances since certain 3D symmetry properties are not satisfied^7^,^8^. The equivalent response from planar metasurfaces can be achieved by alternate methods such as the interaction of plasmon modes^8^,^9^, or by non-radiative dissipation^10^, and is named optical chirality. The difference in transmission from different polarization states due to optical chirality produces an *equivalent* effect to OA and CD^11^.

Optical chirality in metamaterials is produced in several ways. Not surprisingly, a chiral metamaterial can be composed of chiral meta-atoms, in which the mirror image of the structure cannot be superimposed on the original^12^,^13^. Intrinsically chiral materials are chiral due to the geometry of the meta-atoms themselves, and exhibit chiral behavior at normal illumination incidence. Helices^14^, gammadions^15^,^16^, or nanoparticle assemblies^9^,^17^ are common examples of intrinsically chiral meta-atoms that exhibit a strong chiral response. Another approach that achieves chiral asymmetry is the creation of a linear phase gradient, *i.e.*, illumination at oblique incidence^18^,^19^,^20^,^21^,^22^ or spatial variation of the unit cell^5^,^2^,^23^,^24^. The chiral response that arises from the linear phase gradient of either of these approaches is associated with extrinsic chirality.

In this Letter we instead focus our investigation on the optical chirality that arises solely from the interactions between achiral meta-atoms. We postulate that the nonlinear coupling between meta-atoms originates from an interaction force derived from the Liénard-Wiechert potential^25^, which accurately predicts measured changes in the experimental transmission spectra. We study rectangular plasmonic resonators whose arrangement leads to chiral phenomena at normal incidence, or identical tilted achiral nanostructures in a lattice that lead to a chiral response. The individual nanostructures are achiral, yet the periodic array is chiral^26^,^27^, and thus any chiral response distills the interaction between plasmonic structures. Optical chirality is expected when the lines of mirror symmetry of the nanostructures do not coincide with the lattice array —the mirror image cannot be superimposed with the original. “Meta-optical chirality” refers to the macroscopic optical chirality that is both observed in the far-field and attributed to the interactions between plasmonic resonators. While many have alluded to the coupling between meta-atoms, few have addressed the origin of the interaction force.

Experimentally, we employ a simple Babinet-inverted rod (dimensions ≈*λ*/5) as the meta-atom of our metasurface and arrange the nanorods in a square array. When rod-shaped nanoapertures are tilted at an angle of 22.5° and illuminated with low-intensity visible light (≪1 *W*/*cm*^2^), we measure CD on the order of 0.6 degrees of ellipticity. In comparison, while optimized and twisted split ring resonators can reach CD up to 16 degrees in the near infrared^28^, such meta-atoms require small features (≈*λ*/20). The relatively-large dimensions of the rod nanoapertures support dipolar plasmon resonances and can be fabricated easily with robust large-area processes. Herein we demonstrate an approach that leverages the interactions between simpler meta-atoms in order to achieve an optical chirality. The general approach boasts facile design, circumvents the complex structures that intrinsically chiral materials generally require, and forgoes the oblique illumination angle-of-incidence that is the crux of extrinsically chiral materials.

The chiral response exhibited by our metasurface is both appreciable and unexpected since plasmonic interactions generally manifest as nonlinear optical responses, which require high illumination intensities^29^,^30^; here we observe optical chirality at intensities far below those generally required to yield nonlinear optical responses. We interrogate the metasurface with a continuum of polarization states and find that we cannot characterize the metasurface simply from its response from orthogonal circular-polarization modes. In fact, we find that a conventional transmission matrix description of the polarization properties is insufficient to accurately describe the optical behavior of the metasurface. An alternative analytic representation is provided and supports a description of intensity-independent, weakly-nonlinear plasmonic limit cycles. The model also explains why we observe the emergence of multiple eigen-polarization modes, which we also measure experimentally. This work furthers our understanding of metasurface design and breaks from the long-standing convention of transmission matrices.

## The coupling force

In our model, we define the interaction between neighboring plasmonic resonators as the Lorentz force produced from the oscillation of adjacent resonators. The interaction force is derived from the electromagnetic field of an accelerating charged particle given by the Liénard-Wiechert potential^25^. The force from an *m*^*th*^ charge on the *n*^*th*^, 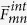, is:


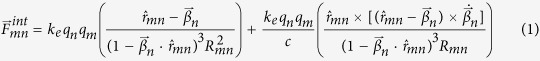


where *k*_*e*_ is Coulomb’s constant =1/4*πε*_0_, *ε*_0_ is the permittivity of free space, 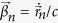, *c* is the speed of light, 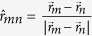 is the unit direction from the *m*^*th*^ charge to the *n*^*th*^, *R*_*mn*_ is the lattice distance, *q*_*n*_ is the charge of the *n*^*th*^ particle, and 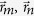 are the positions of the *m*^*th*^, and *n*^*th*^ resonator, respectively, from the origin. When the charges are stationary, Eq. 1 collapses to the Coulomb force between two charged particles. The first term of Eq. 1 is referred to as the “velocity field” since it is independent of acceleration, and the second term is the “acceleration field”. With motion of the charges restricted to the 2-D plane of the metasurface, the interaction forces created by the surrounding resonators are produced in the plane of the metasurface. Though charges also oscillate in the *z*-direction, an investigation into the coupled behavior of the longitudinal fields is beyond the scope of this study. The associated Lorentz forces may explain the near-to-far-field coupling^31^.

**Figure 1:**
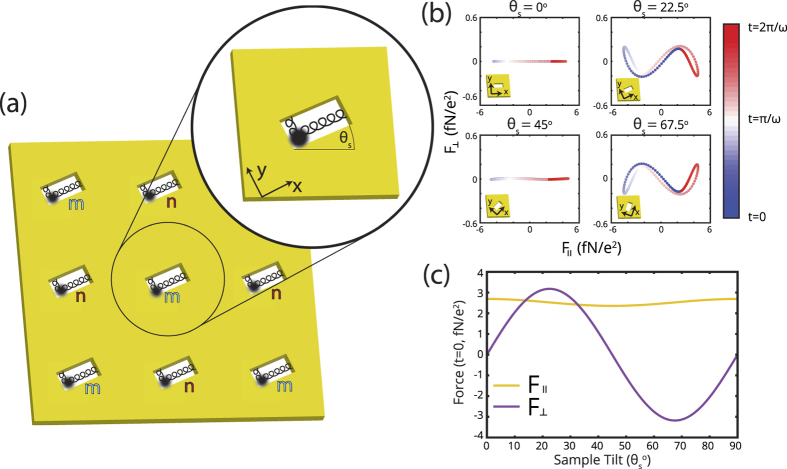
(**a**) Model representation: an array of plasmonic resonators (rod-shaped apertures) tilted at *θ*_*s*_ relative to the base of the unit meta-atom where we separate the resonators into *m*- and *n*-type. The interaction force on *m*-type resonators results from the 4 nearest *n*-type resonators and vice-versa. (**b**) The interaction Lorentz force that arises from Larmor radiation [Eq. 1] for various *θ*_*s*_ when resonators oscillate in the *x*-direction, as a function of time. (**c**) The interaction force in the parallel (*x*), and perpendicular (*y*) directions when the resonators oscillate in the *x*-direction, as a function of *θ*_*s*_.

A representation of the model is shown in Fig. 1(a), in which we tilt all nanostructures by *θ*_*s*_ relative to the base of the unit cell. The tilt effectively restricts the direction of motion of the resonator and we incorporate the interaction force into the equations of motion for a Lorentz-Drude plasmonic resonator^32^. We model metasurfaces in which there is no spatial variation of the unit cell, *i.e.*, all nanoapertures are tilted at the angle, *θ*_*s*_. For analytical reasons the *x* and *y*-axes also rotate by *θ*_*s*_ such that the long (short)-axis of the plasmonic resonator is always parallel with the *x (y*)-axis. The *θ*_*s*_ determines the directions of 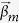, and 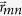 in Eq. 1. We place the resonators in a checkerboard-like pattern of *m*- and *n*-type oscillations, and calculate the motion of each set of resonators. The second-order terms cancel by symmetry, and subsequently the interaction force between resonators scales inversely with the distance cubed. The four closest resonators provide the interaction force that couple motion in the *x*- and *y*-directions, and between *m*- and *n*-type resonators. Interaction forces from diagonal dipoles are between like-type resonators (*m*-*m, n*-*n*) and are neglected, in part because the increased distance between resonators reduces the interaction force, and also because we focus on the interactions between *m*- and *n*-type.

The total interaction force from the Larmor radiation of the four closest resonators is shown in Fig. 1(b,c), where 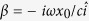 and *x*_0_ is the maximum displacement of the charge oscillation, where we let *x*_0_ = 20 nm. Each resonator oscillates in the *x*-direction and produces a force in both parallel (*x*) and perpendicular (*y*) directions, *F*_||_ and *F*_⊥_. We calculate that *F*_||_ is approximately 20 times larger than *F*_⊥_. The *F*_||_ —which is always co-aligned with the long axis of the nanostructures— is maximal when the nanostructures are oriented at 0° or 90° with the edge of the unit cell. *F*_||_ couples the parallel motion between adjacent resonators in the formation of hybrid modes, which we document in the next section. When the angle of the nanostructure, *θ*_*s*_, is 0°, 45°, or 90°, *F*_⊥_ is zero, as shown in Fig. 1(b,c). These angles correspond to the lines of symmetry of the square lattice; the array is not intrinsically chiral. Alternatively, the magnitude of *F*_⊥_ is maximized at 22.5°, and 67.5° which would correspond to opposite-handed chiral structures. *F*_⊥_ couples to the orthogonal dipole moment of the adjacent resonators and is the source of optical chirality in our metasurface, which is further documented in the following section. Though our model incorporates the Liénard-Wiechert potential from rectangular nanostructures, the model can be generalized to model the interaction forces from arbitrary plasmonic shapes and arrays.

## Coupled equations of motion

We sum the interaction force [Eq. 1] from the four neighboring resonators to the Lorentz-Drude model and incorporate its interaction as a perturbation with the parameter, *δ*: 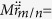

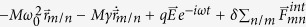
, where 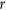 is the displacement of the resonator from its equilibrium position, 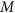 is its mass, 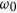 is the natural harmonic frequency of the resonator, 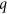 is its charge, 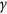 is the velocity-dependent damping rate, 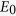 is the strength of the incident driving electric field, with frequency 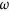, subscript 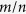 refers to the motion of either 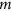
- or 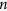-type resonators, and dot formalism corresponds to derivatives with respect to time, 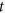. We adopt a separate solution for the displacement of the 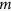
- and 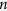-type resonators shown below:


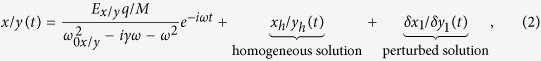


which includes a homogeneous solution, *x*_*h*_, *y*_*h*_, often neglected^29^,^30^,^31^. The homogeneous solution is the general solution to the equations of motion in the absence of an external electric field and its solution is determined from the initial conditions. Even though the homogeneous solution is neglected in Optics texts, it is generally present in studies of vibrations and when solving for the limit cycles of weakly nonlinear oscillators^34^. No exact analytical solution exists for the system in which the interaction force is included; our quantitative perturbative approach employs a Taylor expansion of the interaction force,


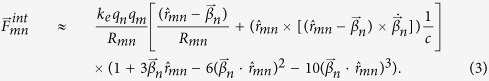


We consider the low-power case where the transmitted polarization is independent of illumination intensity and we linearize the interaction force with the electric field. The system remains nonlinear because the optical response cannot be described by a superposition of incident fields.


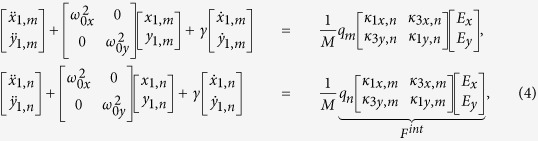


where


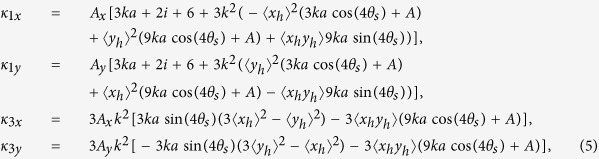


and where 
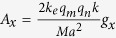
, 
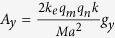
, *A* = 3*ka* + 4*i, a* is the periodicity of the metasurface, *k* is the incident wavevector, 〈〉 denotes the time-average, and 
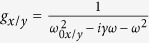
.

While Eq. 4 may resemble a transmission-matrix formalism, the equations of motion of one type of resonator are coupled to the other by its homogeneous solution and subsequently, there are four coupled equations of motion instead of two. When the *m*-type resonators are excited, they generate Lorentz forces via the Liénard-Wiechert potential, which contribute to both parallel and perpendicular motions of the *n*-type resonators.

The perpendicular Lorentz force couples to the orthogonal modes of the different resonators, which leads to optical chirality in the metasurface. In order to understand the terms of Eq. 5, we isolate the case where the plasmonic resonators move in either the *x*- or *y*-directions in the fundamental solution. The perturbative interaction becomes 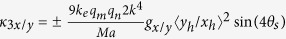, which is zero when *θ*_*s*_ = 0°, 45°, 90°, *etc.*, in agreement with the exact evaluation of *F*^*int*^ [Fig. 1(b,c)]. Maximal optical chirality is achieved when the nanostructures are tilted at *θ*_*s*_ = 22.5° and 67.5°.

## Mode hybridization and optical chirality

**Figure 2:**
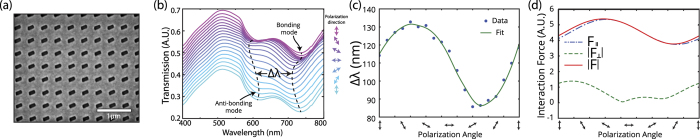
(**a**) A top-view scanning electron microscope image of the metasurface. (**b**) The transmission spectra associated with different linear polarization angles, where we observe the presence of bonding and anti-bonding modes. (**c**) Shift in the wavelengths of the bonding and anti-bonding modes as a function of polarization angle. (**d**) Interaction forces parallel and perpendicular to the polarization axis experienced by the resonators from Eq. 4 as a function of polarization angle.

Experimentally, we fabricate a metasurface that maximizes *F*_⊥_ or *κ*_3*x*/*y*_ with *θ*_*s*_ = 22.5°, in which rod nanoapertures are arranged on a 30-nm gold film and measure 160 nm by 80 nm, as shown in Fig. 2(a). The periodicity, *a*, varies the relative strength of the interaction force. We utilize a periodicity of 375 nm in a square array, which is small enough to observe the coupling between nanoapertures, yet large enough so that the individual responses are still present.

The wavelength-dependent transmission properties are explored in Fig. 2(b), which shows the transmission for linearly-polarized light. The axis of the illuminating linear polarization is rotated counter-clockwise from 0° (vertical) through 90° (horizontal) to 180° (vertical). We observe two absorption dips in the transmission around 600 nm and 730 nm that correspond to the mode-splitting or mode hybridization of the plasmonic dipolar resonance^35^. The spectral locations of the bonding and anti-bonding modes correspond to the mode coupling or interaction force *F*^*int*^. When the periodicity decreases, *F*^*int*^ increases and the resonances separate further; if the separation between meta-atoms increases, then the transmission resonances collapse to the transmission profile of a single resonator^35^. The trends associated with meta-atom spacing indicate that the transmission dips observed are not a Wood’s anomaly grating effect, which is related to lattice absorption. Wood’s anomaly resonances would scale in proportion with the metasurface spacing. Moreover, Wood’s anomalies are characterized by ultrasharp resonances^36^ which are not observed.

The parallel component of the interaction force leads to the mode-splitting in the metasurface. The spectral difference between the bonding and anti-bonding modes is shown in Fig. 2(c) with a Savitzky-Golay filter^37^ overlaid. We evaluate the interaction force in our approximation [Eq. 4] in order to relate our model to the mode splitting. The components of the interaction force, *F*_⊥_, *F*_||_, and 
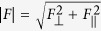
 are shown in Fig. 2(d), where the input polarization is rotated from 0° to 180°. The *F*_||_ and the trendline of the spectral shift both exhibit maximal values between 45° and 55°, where the illuminating linear polarization connects opposite ends of vertically-adjacent resonators. The minimal values of the spectral splitting occur between 135° and 145° and correspond with an angle where the illuminating linear polarization connects opposite ends of horizontally-adjacent resonators. Differences between our model and experiment result from the fact that in our model, we only consider the resonator displacement of 20 nm whereas in experiment, the effective resonator dipole is the length of the nanorod, or 180 nm. Moreover, our approximation of *F*^*int*^ only accounts for the four nearest resonators and incorporates only time-harmonic terms. Nevertheless, the strength of the parallel force *F*_||_ scales approximately with the experimentally-measured spectral splitting of transmission resonances.

**Figure 3:**
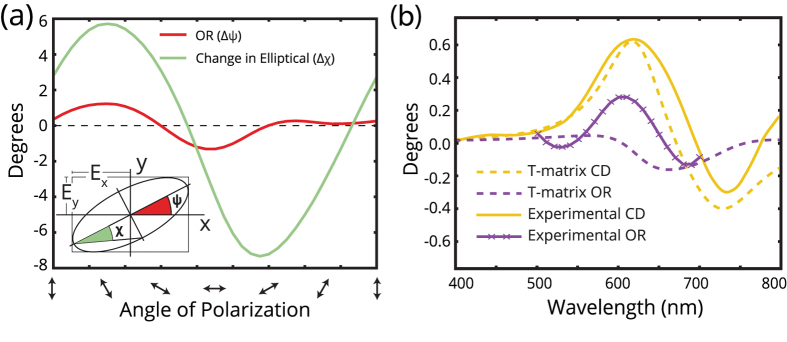
(**a**) The optical rotation (OR) and change in the elliptical component as a function of input polarization at 660 nm. Inset: the relationship between *E*_*x*_ and *E*_*y*_ OR and change in the elliptical on the polarization ellipse. (**b**) OR and circular dichroism (CD) calculated from a transmission (T-)matrix approach (dashed). Overlaid is the experimental measurements of OR (measured at *ψ* = +30°), and the CD (solid).

The interaction force leads to other experimentally-measured polarization-dependent metasurface responses. We illuminate the metasurface with linearly-polarized light (ellipticity *χ* < 1°) at a wavelength *λ* = 660 nm and observe that the transmitted changes in the azimuthal and elliptical components of the fields, Δ*ψ* and Δ*χ*, depend on the angle of linear polarization [Fig. 3(a)]. The change in azimuth Δ*ψ* denotes optical rotation (OR) and the change in the elliptical component Δ*χ* denotes the phase accumulation between the *E*_*x*_ and *E*_*y*_-components. When the illuminating polarization angle rotates a full revolution, the trendline for the OR exhibits 4 inflection points. The OR and the perpendicular interaction force *F*_⊥_ follow a similar trend [Fig. 2(d)], which is expected since *F*_⊥_ is the source of the optical chirality in our metasurface.

Optical chirality is conventionally determined by the off-diagonal elements of transmission matrix of the metasurface^26^,^38^,^39^ but there are limits to this transmission matrix formalism. The polarization density of the material is calculated with the relation 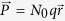, where *N*_0_ is the free charge carrier concentration of the material, *q* is the charge of the particles, and 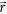 is the direction and subsequently: 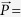

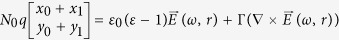
, and the non-locality parameter, 
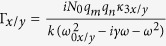
, and Γ is the mean of Γ_*x*_ and Γ_*y*_. The relations for 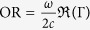, and 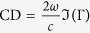, are shown as a function of the incident wavelength for tilt angle of *θ*_*s*_ = 22.5° in Fig. 3(b) and the experimental observation is overlaid. The CD is defined as the differential circular-polarization absorption as CD(^*o*^) = (*T*_*RCP*_ − *T*_*LCP*_) × 32.982° in degrees of ellipticity^40^. The experimental results for CD qualitatively agree well with the analytical theory for the CD, where peaks in CD are present at both dipolar modes. In a transmission-matrix calculation, there is less agreement between the real component of Γ and the experimentally-measured OR. We expect that CD may be more accurately modeled with transmission matrices because the rotating circular polarization averages all linear-polarization responses, and reduces the polarization-dependence illustrated in Fig. 2. The discrepancies between theoretical and experimental OR indicate the limit of the transmission matrix formalism that assumes that all resonators oscillate in-phase, which may not be true when an interaction force is present between meta-atoms.

## Emergent polarization eigenmodes

**Figure 4:**
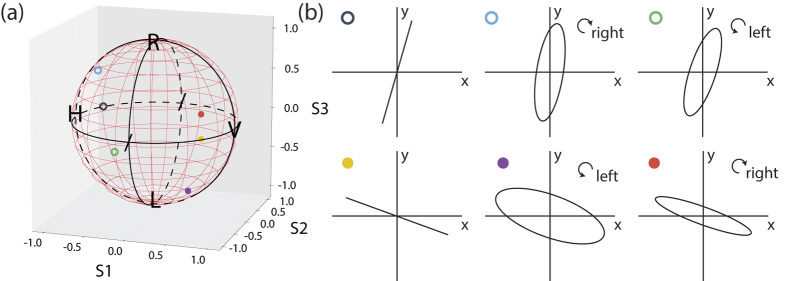
Eigen-polarization modes of the system at 660 nm are shown on (**a**), the Poincaré sphere, where solid circles represent eigen-polarization states on the near surface of the sphere and hollow circles represent states on the far surface. (**b**) illustrates the eigen-polarization states measured in the *x* − *y* plane.

We interrogate the metasurface with a continuum of polarization states and observe polarization-dependent responses other than OR that cannot be recreated by transmission matrices. We characterize the properties of the metasurface eigen-polarization modes at a wavelength of 660 nm on the Poincaré sphere [Fig. 4(a)]. Figure 4(b) shows the eigen-polarization modes mapped onto the *x* − *y* plane, where the wave travels in the *z*-direction. The eigen-polarization modes of the system are interpreted as eigenmodes of the coupled equations of motion of the system.

We measure six eigen-polarization modes of the metasurface, where a standard transmission matrix approach asserts the existence of only two^41^. Two of the six eigen-polarization modes are approximately linearly-polarized, and the remaining four are elliptically-polarized. The presence of the multiple eigen-polarization modes cannot be characterized by the conventional, single transmission matrix formulation —which assumes that adjacent resonators are excited in phase —and supports an alternative model of coupled resonators.

There is potentially a misconception that transmission matrices should describe any metasurface optical response that is independent of the illumination intensity. The optical response from our metasurface is linear because the transmitted polarization does not change with illumination intensity and would not produce the prototypical nonlinear optical response, for example, higher-harmonic generation, particularly at low illumination intensities. At the same time, the transmitted polarization from our metasurface is nonlinear in a manner that the optical response cannot be described by a superposition of incident fields. It appears insufficient to characterize the polarization properties of the metasurface by deriving a transmission matrix from the optical response of select linear or orthogonal circular polarizations. The corresponding system of equations in our model predicts multiple polarization eigenmodes that are observed at visible wavelengths and at low illumination power, independent of illumination intensity.

## Discussion and Conclusion

The inclusion of an interaction force leads to responses that are not predicted via transmission matrices, such as polarization-dependent OR or multiple eigen-polarization modes, which we measure in this investigation. Discrepancies from a transmission matrix formalism may be minimal when the interaction force is small and when meta-atoms are separated significantly far apart^42^. However, initial calculations suggest that the Larmor radiation from adjacent meta-atoms achieves intensities comparable to that of the incident electric field, even at low illumination intensities, due to the strong acceleration of charges on sub-wavelength nanostructures at visible wavelengths. Moreover, the constructive interference of Larmor fields in periodic lattice structures such as metasurfaces is measurable. The Larmor radiation field may be engineered to produce a desired electromagnetic response. One method to increase the Larmor radiation, and thus the optical chirality, is to reduce the periodicity of the nanostructures since the interaction force [Eq. 1] scales with the inverse distance between resonators. However, as Lee *et al*.^43^ showed, the dependence on periodicity does not continue to increase the interactions forces indefinitely since at the limit of zero periodicity we expect the scattering cross section of the array of coupled plasmonic resonators to reduce to that of a single resonator. In order to satisfy this condition, a screening factor of 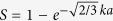 is employed so that the interaction forces converge to zero at zero periodicity.

In conclusion we have proposed a facile design approach to achieve optical chirality at normal incidence in planar arrays of achiral nanoapertures that leverages the interaction force between coupled resonators. Optical chirality results from perpendicular Lorentz forces associated with the Liénard-Wiechert potentials when plasmonic resonators are sufficiently close. Bonding/anti-bonding modes observed in the transmission spectra result from the parallel components of the interaction forces. We have studied the polarization properties and demonstrated that our metasurface exhibits both OR and CD equivalent responses at normal incidence in the visible regime, a property found only in chiral materials. The OR depends on the incident angle of polarization and cannot be fully described by transmission matrices. We further demonstrate this principle through the measurements of several eigen-polarization modes. The presence of appreciable optical chirality in our metasurface indicate Larmor radiation plays a significant role in the polarization properties of metasurfaces.

## Methods

The periodic array of rod nanoapertures is fabricated with top-down methods. On a glass substrate a 5-nm wetting layer of chromium followed by a 30-nm layer of gold is deposited via electron beam deposition. Following, a 300-nm layer of ZEP 520a resist is spin-coated on top. A 1.5 mm × 1.5 mm area is patterned onto the substrate with electron beam lithography. The resist is developed with hexyl acetate at −25 °C for high contrast. Finally, the gold is etched away with ion beam milling. Figure 2(a) shows a scanning electron microscope image of the metasurface from the top.

**Figure 5:**
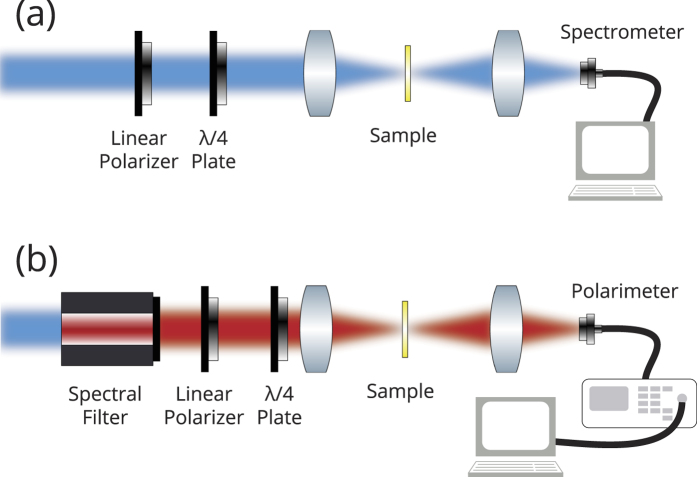
The schematic diagram for (**a**) transmission measurements and (**b**) polarization measurements.

To measure the optical chirality from the metasurface we use a xenon solar simulator that provides stable broadband illumination between 400 nm and 1000 nm. The polarization state of light is controlled with a wire grid polarizer a 400-nm to 800-nm achromatic quarter-wave plate. The spectrum from the sample is captured via a CCD-coupled spectrometer[Fig. 5(a)].

The eigen-polarization states are determined in a similar setup described above, however a spectral filter is used and the CCD-coupled spectrometer is replaced with a polarimeter to determine the polarization of transmitted light[[Fig. 5(b)]].

## Additional Information

**How to cite this article**: Moocarme, M. *et al*. Meta-Optical Chirality and Emergent Eigen-polarization Modes via Plasmon Interactions. *Sci. Rep.* **7**, 40718; doi: 10.1038/srep40718 (2017).

**Publisher's note:** Springer Nature remains neutral with regard to jurisdictional claims in published maps and institutional affiliations.
